# A putative siderophore receptor of *Gallibacterium anatis* 12656-12 under Fur control also binds hemoglobin

**DOI:** 10.3389/fmicb.2022.951173

**Published:** 2022-08-16

**Authors:** Alberto Chantes-Guerra, Samantha Maldonado-Puga, Norma Rojas-Ruiz, Ismael Rea-Hernandez, Fernando J. Montes-Garcia, Hector Trujillo-Ruiz, Ivan E. Yañez-Aguilar, Candelario Vazquez-Cruz, Patricia Sanchez-Alonso, Erasmo Negrete-Abascal

**Affiliations:** ^1^Instituto de Ciencias, Benemérita Universidad Autonóma de Puebla, Puebla, Mexico; ^2^Centro de Investigaciones y Estudios Avanzados, Instituto Politécnico Nacional de México (CINVESTAV), Mexico City, Mexico; ^3^Facultad de Estudios Superiores Iztacala, Universidad Nacional Autonóma de Mexico, Los Reyes Iztacala, Tlalnepantla de Baz, Edo de México, Mexico; ^4^Científicos Veterinarios de México, Tepatitlán, Mexico

**Keywords:** *Gallibacterium*, Fur, iron restriction, *G. anatis fur*-mutant, biofilm, virulence

## Abstract

*Pasteurellaceae* family members obtain iron directly from host proteins or through siderophore-dependent mechanisms. Although *Gallibacterum anatis* expresses different virulence factors, its response to growth under iron restriction is unknown. *G. anatis* cultured in the presence of 2,2′-dipyridyl, up-expressed an approximately 65 kDa protein and repressed the expression of a 70 kDa protein. MALDI-TOF analysis of those proteins indicated homology with CirA (65 kDa), a protein involved in iron-siderophore acquisition in *Mannheimia succinoproducens* and a TonB-dependent receptor (70 kDa protein), a protein that binds chicken hemoglobin; however, *G. anatis* siderophore production was not detected by chromo azurol S (CAS)-BHI agar determination. This putative *G. anatis* siderophore receptor is under Fur control, but not the hemoglobin binding protein, as observed in *G. anatis* 12656-12 *fur* mutant (Ω *fur* 126.13) grown in the presence or not of 2,2′-dipyridyl. The addition of FeCl_3_ to the culture medium diminished the growth and biofilm production in approximately 30% and 35%, respectively, in the wild-type strain, but the growth of Ω *fur* 126.13 strain was not affected and biofilm production increased in 35%. *G. anatis* Ω *fur* 126.13 presented lower virulence when it was inoculated to 35-day-old chickens in comparison to the wild-type strain. The induction of more than one iron uptake mechanism could benefit pathogenic microorganisms such as *Gallibacterium*.

## Introduction

*Gallibacterium* is a genus of the *Pasteurellaceae* family (Christensen et al., 2003). This genus is composed of different species, of which only *Gallibacterium anatis* has been associated with chickens (Bisgaard et al., 2009). This microorganism has been considered part of the autochthonous microbiota of the respiratory and lower genital tracks of chickens; however, it has also been associated with different pathologies, including salpingitis, peritonitis, and oophoritis (Neubauer et al., 2009). Lesions of the reproductive organs are responsible for a reduced egg production and increased mortality of chickens, which cause economic losses to the poultry industry worldwide (Bojesen et al., 2008).

Few virulence factors have been described to be expressed by this bacterium: chicken IgG-degrading secreted proteases (García-Gómez et al., 2005), expression of a RTX (Repeat in toxin) protein (Kristensen et al., 2010), as well as the ability to agglutinate red blood cells (Zepeda et al., 2009; Montes-García et al., 2016), and the ability to adhere to plastic surfaces and to form biofilms on glass (Vaca et al., 2011; Persson and Bojesen, 2015). This last capability could be supported by the fimbriae expression described recently (Salgado-Lucio et al., 2012; Bager et al., 2013; Kudirkiene et al., 2014; López-Ochoa et al., 2017) or by the participation of other molecules as putative adhesins like the EF-Tu protein (López-Ochoa et al., 2017). However, the response to different stresses and the putative relationship with virulence factors expression have not been studied.

Iron is an elemental microelement necessary for most microorganisms (Krewulak and Vogel, 2008). Different bacteria can acquire iron from host molecules by expressing outer membrane receptors for transferrin or hemeproteins and by producing or using siderophores from other microorganisms; the expression of most of these receptors is controlled by the ferric uptake regulator (Fur) protein. Fur protein is responsible for controlling the acquisition and transport mechanisms of iron ions in different bacteria (Jin-Won and Helmann, 2007; Krewulak and Vogel, 2008). In the present work, we identified two iron restriction-related proteins, a putative siderophore receptor under Fur control, induced in response to a lack of iron and a hemoglobin-binding protein that is not Fur-regulated when *G. anatis* is grown in the presence of 2,2′-dipyridyl as an iron-chelating agent. In addition, we describe the construction of a *G. anatis* 12656-12 mutant *fur* gene obtained by interruption of this gene with a streptomycin cassette using an integration vector recently described (López-Ochoa et al., 2019). Furthermore, the effect of *fur* interruption was evaluated by comparing the biofilm formation capability with respect to the *G. anatis* 12656-12 wild-type. Both strains were also evaluated with a virulence bioassay in chickens. Results obtained suggest that different iron uptake and control mechanisms could confer advantages to this avian pathogenic microorganism to grow within its infected host.

## Materials and methods

### Bacterial strain and growth conditions

*G. anatis* 12656-12 hemolytic, virulent and F149^T^ non-hemolytic and non-virulent varieties were grown in brain heart infusion (BHI) medium for 24 h at 37°C under agitation. *E. coli* DH5α was used to made constructs. *E. coli* and *Pseudomonas aeruginosa* PAO1 were cultured in LB-broth to test siderophores production. *G. anatis* F114 and ESV34 strains hemolytic varieties (Montes-García et al., 2016) were also included in this study. Several *fur*-mutant strains were obtained in this work, but the mutant assayed *in vivo* here was Ω *fur* 126.13.

To eliminate the iron from the glassware, the material was soaked overnight in 0.5% ethylenediaminetetraacetic acid (EDTA) and then washed with deionized water, as previously described (Abascal et al., 2009); deionized water was used for the preparation of all solutions and culture media.

To evaluate the effect of iron on protein expression, an overnight culture of *G. anatis* was diluted 1:100 in flasks containing fresh BHI and incubated at 37°C under shaking until the cultures reached 0.1 optical density units at 600 nm. Next, the specific iron-chelating agent, 2,2′-dipyridyl (Sigma Aldrich, St Louis, MO, United States), was added at 0.25 mM (final concentration), keeping one flask as a control without addition. All assays were done in triplicate at least 3 times.

### Collection of outer membrane proteins

*G. anatis* was cultured for 24 h in a 500-mL flask containing 250 mL of BHI supplemented with 2,2′-dipyridyl as above. Cells were separated by centrifugation (10,500 *g* during 30 min at 8°C) and suspended in 5 mL of 20 mM HEPES, pH 7.4, and lysozyme (1 mg/ml). Samples were sonicated on ice for seven cycles of 10 s on/10 s off each. After sonication, samples were processed as described previously (Abascal et al., 2009). The protein pellet was suspended in 500 μL of 20 mM HEPES supplemented with 1% Triton X-100 (final concentration, Thermo Fisher Scientific, Waltham, MA, United States). Triton-soluble or insoluble proteins were evaluated. Protein concentration was determined by the Bradford method using bovine serum albumin as standard (Bradford, 1976).

### Sodium dodecyl sulfate-polyacrylamide gel electrophoresis

To identify proteins expressed by bacteria cultured in iron-restricted or in normal medium, samples containing 15 μg per well were loaded and were electrophoretically separated by 10% sodium dodecyl sulfate-polyacrylamide gel electrophoresis (SDS-PAGE). After electrophoresis, gels were stained with Coomassie blue R.

### Peptide mass fingerprint

The peptide mass fingerprints of the selected outer membrane proteins (OMPs) were determined by matrix-assisted laser desorption ionization time-of-flight mass spectrometry (MALDI-TOF MS) after trypsin digestion of the proteins identified through SDS-PAGE (Abascal et al., 2009).

### Siderophores

To search for siderophore production, *G. anatis* 12656-12 and *Pseudomonas aeruginosa* were grown in M9 minimum medium with 0.1 g/L polypeptone and 0.15 g/L casamino acids. Cultures were incubated under agitation for 48 h at 37°C in the presence or absence of 0.25 mM 2,2′-dipyridyl. Cultures were centrifuged as above, and cell-free culture supernatants were filtered through a 0.22-μm nitrocellulose membrane. The spent medium (2 mL) was mixed with the same volume of a 2% (w/v) FeCl_3_ solution, and the production of siderophores was assayed by spectrophotometric measurements from 400 to 600 nm according to Neilands (1981). Siderophore production was also evaluated on chrome azurol S (CAS)-BHI agar as described by Tabatabai et al. (2008) using *P. aeruginosa* O1 and *Escherichia coli* DH5α as positive and negative (Chart et al., 2000) siderophore-producer strains, respectively.

### Chicken hemoglobin procurement

Chicken hemoglobin was obtained according to Drabkin (1946). Briefly, 0.9% NaCl (final concentration) was added to 200 mL of chicken blood. Red blood cells were collected by centrifugation at 2,000 rpm for 5 min. Chicken erythrocytes were lysed with distilled water (2 volumes), and membrane cells were removed in the same conditions. Chicken hemoglobin was recovered by precipitation with 35%, 50%, and 70% ammonium sulfate. Proteins recovered with 70% ammonium sulfate were dialyzed against PBS and lyophilized for storage. Chicken hemoglobin was dissolved and biotinylated as previously described (Kittigul et al., 1998).

### Immune recognition

To evaluate the immunogenicity of stressed and unstressed cultures of *G. anatis*, extracted proteins were separated by 10% SDS-PAGE and transferred to a nitrocellulose membrane, as described previously, to determine proteins over- or under-expressed in the iron-restricted medium (Abascal et al., 2009). In this assay, membranes were incubated under one of the three following conditions: with a 1:1,000 diluted polyclonal serum pooled from chickens experimentally infected with *G. anatis* (Salgado-Lucio et al., 2012), anti-transferrin binding proteins (TbpA and TbpB) from *Avibacterium paragallinarum* (Abascal et al., 2009), or biotinylated-chicken hemoglobin. Next, the membranes were washed three times with phosphate-buffered saline-0.5% Tween 20. The immune reaction was revealed with rabbit IgG anti-chicken antibody, goat IgG anti-rabbit antibody or avidin, all of which were peroxidase- labeled, using diaminobenzidine and H_2_O_2_ as substrates.

### *G. anatis* 12656-12 locus *fur* amplification and cloning

**FIGURE 1:**
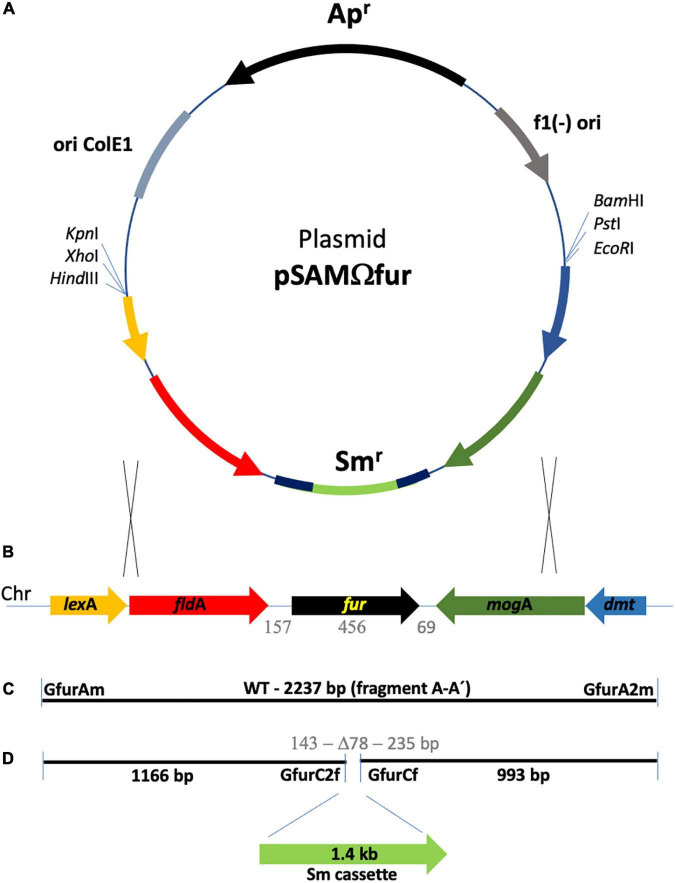
Schematic representation of the *fur* locus in the *G. anatis* 12656-12 genome and strategy of its disruption. **(A)** Diagram of plasmid pSAMΩfur, the one-step disruption cassette contains a streptomycin resistance gene flanked by regions of homology shown in red (left) and green (right). **(B)** Map of the *fur* locus located on the chromosome of *G. anatis* 12656-12*; lexA, fldA, fur, mogA*, and *dmt* are represented as arrows. The genes are homologous to those held in loci UNM179_R10075 to UMN179_R10095 from strain *G. anatis* UMN179 (accession NC_015460.1). **(C)** The A-A’ fragment is a PCR amplification product of 2,237 bp, using GfurAm and GfurA2m primers. The gray numbers indicate the size in base pairs of *fur* and adjacent intergenic sequences. **(D)** The sequences flanking the deletion site at the *fur* locus are 1,166 and 993 bp in size and were ligated to a 1.4 kb streptomycin cassette as part of the plasmid shown in A. pSAMΩfur is an integration plasmid that contains the *col*E1 replicon but is unable to replicate in *G. anatis*; therefore, resistance to streptomycin will only be possible after integration of the cassette into the chromosome. Replacing the *fur* WT locus with the one-step disruption cassette is indicated by two pairs of crossed lines; the interrupted A-A’ fragment increases the size of the locus by 1.4 kb. The gray numbers indicate the 78 bp deletion and, on the sides, the size of the remaining *fur* DNA in the construct.

To amplify a DNA fragment containing the *G. anatis fur* gene (593 bp), oligonucleotides GfurAm (ccacaaaacggcttatcaatctca) and GfurA2m (ttaatttatatgccggtgcgtgaa) were designed based on the genomic sequencing data (NC_015460.1). They were designed considering the left and right fragments flanking the *G. anatis* UMN179 *fur* gene sequence (Figure 1) to amplify a 2.2 kbp fragment and increase the possibility to obtain a double crossover recombination on this fragment when *G. anatis* cells were transformed (Kristensen et al., 2012). *G. anatis* 12656-12 genomic DNA was obtained according to Sambrook et al. (1989), and the *fur* locus was amplified by polymerase chain reaction (PCR) under the following conditions: 94°C, 5 min; 94°C, 30 s, 55.5°C, 30 s, and 72°C, 2.5 min (28 cycles); and 72°C, 10 min. The PCR amplification product was visualized in a 0.8% agarose gel and cloned into the pBluescript KS II(-) vector (Stratagene, Kirkland, WA, United States), generating the plasmid pSAMfur. Plasmid DNA was purified using the Wizard Plus SV Miniprep DNA Purification System (Promega, Madison, WI. United States) and sequenced in a Perkin-Elmer analyzer (ABI PRISM^®^ 3100 sequencer) using M13 reverse and forward universal primers. The sequence obtained was analyzed through the BLASTn, BLASTp^1^, and ClustalW^2^ programs.

### *G. anatis fur* gene interruption

Once the presence of the *fur* gene in the *locus* (2.2 kb) cloned in pSAMfur was confirmed, an inverse PCR to get the *fur* gene interruption was performed to open the construct under the following conditions: 94°C, 5 min; 94°C, 30 s, 47°C, 30 s, and 72°C, 5 min (28 cycles); and 72°C, 10 min using primers GfurCf (tcgccataattttgaaggaa) and GfurC2f (aaccatttataaacatcttcag) (Figures 1B,C). The amplified product (5.1 kb) was used to clone a streptomycin cassette (1.4 kbp), obtained from a purified plasmid (pOV) from a multi-resistance pig *Pasteurella multocida* isolated and previously characterized (López-Ochoa et al., 2019), using primers Str2L (gatatcgaagggggtagttggt) and Str3U (gatatcatttgccggtgcttctgt) within the *fur* coding sequence, generating the plasmid pSAMΩfur (Figures 1A,D). Plasmid pSAMΩfur was sequenced to verify the construct and was used to transform competent *G. anatis* 12656-12 cells obtained, as described by Kristensen et al. (2012), using M-IV chemical transformation; double recombination events were selected by the streptomycin-resistant phenotype.

### Genomic verification of the *G. anatis* 12656-12 *fur* mutants

Genomic DNA from *G. anatis* 126.13 and 126.5 Ω *fur* strains, as well as pSAMΩfur, and wild-type *G. anatis* cultures was extracted with phenol-chloroform and used to verify the replacement of the chromosomal *fur* gene in *G. anatis* 12656-12 by PCR amplification of the interrupted *fur* locus using primers GfurAm and GfurA2m, as above. This was also performed by DNA-DNA hybridization of genomic DNA digested with *Hind*III restriction enzyme and separated by electrophoresis on an agarose gel (0.8%). Separated DNA was transferred to a nylon membrane (Millipore-Merck, Sigma Aldrich, St. Louis, MO, United States) and hybridized with α^32^P-dCTP-labeled *flavodoxin-fur* (using primers Gmog-tgcgcccgttacttatacc and Gseq-cacctgatgcaacgttacgcc) or streptomycin cassette probes obtained by PCR. The signals representing hybridized DNA were: 4.2 kbp for WT-HindIII-fragment in size and 5.6 kbp for *fur* mutants and were observed by Kodak X-ray film-exposure (Vázquez-Cruz and Olmedo-Álvarez, 1997).

### Effect of FeCl_3_ on *G. anatis* growth and biofilm formation

To know the effect of FeCl_3_ on the growth of *G. anatis* 12656-12 strain and its Ω *fur* 126.13 mutant, bacteria were grown in BHI medium supplemented with 100 μM FeCl_3_ in overnight cultures and determining the OD_600 nm_. The biofilm production by both strains was evaluated, as previously described, in a medium supplemented or not with 100 μM FeCl_3_ (Vaca et al., 2011). Biofilm quantification was determined through OD_595 nm_ lectures of resolubilized Hucker’s crystal violet in 33% glacial acetic acid solution.

### Virulence assay

To evaluate if the inactivation of *G. anatis fur* gene had any effect on the virulence, 35-day-old clinically healthy, specific-pathogen-freeleghorn chickens (SPF, Puebla, Mexico) were used in the study. Chickens were housed in isolators with antibiotics-free food and bottled water *ad libitum*. Groups of 10 chickens were separated in a single isolator and infected by nasal instillation with 0.2 mL of an overnight culture of wild type *G. anatis* or its mutant Ω *fur* 126.13 containing 1 × 10^8^ CFU/mL (Trujillo-Ruíz et al., 2016). One group was instilled with 0.2 mL of PBS as a control. Chickens were examined for 10 days and euthanized after the last examination. Bacterial isolation was attained by sampling infraorbital sinuses and different organs and cultured on BHI blood agar.

### Bioinformatics analysis of TonB-dependent receptors in *G. anatis*

The TonB-dependent receptors were analyzed using as a seed the TonB-dependent receptors included in *ton*B operons from *G. anatis* 12656-12, F149^T^, and UMN179 (AVOX01000000.1, JPHN01000000.1, NC_015460.1). The last one because it is in a circular genome and is a genomic reference. Regulatory sequences were predicted on nucleotide sequences from the TonB-dependent receptor and *fur* genes with Bprom from Softberry^3^. Proteins were analyzed by BLASTp^4^, in single and grouped mode. Alignments were done with Clustal Omega^5^ and the phylogenetic tree was redrawn with iTOL Tree viewer^6^. Amino acid sequences were also compared with 3D models in PDB^7^ and SWISS-MODEL^8^. Prediction of beta-barrel outer membrane protein was done with PRED (http://bioinformatics.biol.uoa.gr/PRED-TMBB) Supplementary Figure and Supplementary Data Sheet.

## Results

### Iron restriction conditions

**FIGURE 2:**
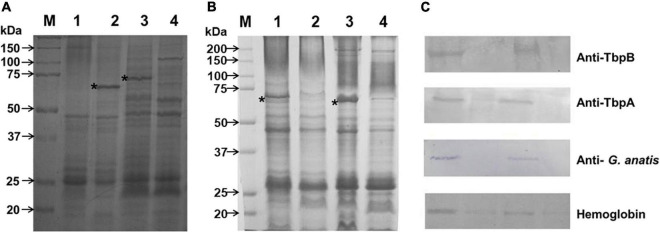
Up and down expression of proteins due to iron depletion. **(A)** Triton-insoluble (lanes 1 and 2) and Triton-soluble (lanes 3 and 4) proteins from *G. anatis* 12656-12 were separated by electrophoresis on 10% polyacrylamide gels and stained as described under Material and methods. The bacterium was grown under iron-restricted conditions (lanes 2 and 4) or normal conditions (lanes 1 and 3); the 65-kDa and 70-kDa proteins are shown in lanes 2 and 3, respectively (*). **(B)** Differential expression of the 65-kDa protein when *G. anatis* F149^T^ (lanes 1 and 2) or 12656-12 (lanes 3 and 4) were grown under iron-restricted (lanes 1 and 3) or normal conditions (lanes 2 and 4). *Indicates 65- kDa protein in Triton-insoluble samples. **(C)** Immunological recognition of the 65- kDa iron-restricted protein from *G. anatis* F149^T^ and 12656-12 by polyclonal sera against TbpB and TbpA proteins, both from *A. paragallinarum*, or by pooled sera from chickens infected with *G. anatis*; or interaction with biotinylated chicken hemoglobin.

The growth of *G. anatis* wild type or Ω *fur* 126.13 strains in the presence of 2,2′-dipyridyl exhibited a reduction of approximately 60% and 37%, respectively, compared with cultures with no addition of 2,2′-dipyridyl. The expression of an up-induced 65-kDa protein (Figure 2A lane 2) or a down-induced 70 kDa (Figure 2A, lane 3) in iron restriction condition was observed in triton-insoluble (lanes 1 and 2) and Triton-soluble (lanes 3 and 4) Omps from *G. anatis* 12656-12, respectively.

### Western and Far western blotting

By polyacrylamide gel electrophoresis, a band of approximately 65 kDa corresponding to a Triton-insoluble OMP was observed in samples of *G. anatis* grown in iron-restricted conditions but not in cultures without the addition of 2,2′-dipyridyl (Figure 2B). This OMP was immune-recognized by rabbit polyclonal antiserum against TbpB and TbpA from *A. paragallinarum*, indicating its relationship with the structure of iron-binding proteins (Figure 2C), or by sera from chickens infected with *G. anatis* (Figure 2C), suggesting its expression *in vivo*. Additionally, this protein was able to interact with biotin-labeled chicken hemoglobin (Figure 2C).

**FIGURE 3:**
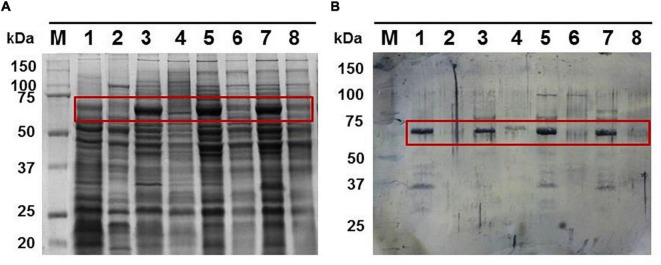
**(A)** Total cell extracts protein pattern in a 10% polyacrylamide gel from different *G. anatis* grown in normal (lanes 1, 3, 5, and 7) or iron-restricted conditions (lanes 2, 4, 6, and 8) of the 12566-12, F149^T^, F114, and ESV34 strains, respectively. The rectangle indicates the hemoglobin-binding protein. Twenty-five micrograms were loaded per well. **(B)** Interaction of proteins with biotinylated chicken hemoglobin.

An approximately 70 kDa OMP, from total cell extracts (Figure 3B), interacted with biotin-labeled chicken hemoglobin in samples of *G. anatis* grown in the presence or absence of 2,2′-dipyridyl, and was expressed at lower levels by *G. anatis* 12656-12 in comparison with the F149^T^ strain (Figure 3B); however, this interaction was more intense in samples from *G. anatis* grown in the absence of the chelating agent (Figure 3B). This protein was also immune-recognized by the serum of chickens infected with *G. anatis*, suggesting that this protein is expressed *in vivo* (Figure 2C).

### OMPs identity

Tryptic digestion of the 65-kDa OMP produced four peptides with a mass-to-charge ratio (M/z) ranging from 440 to approximately 800. The peptide mass fingerprint was compared using the Mascot server^9^. The mass spectrometry analysis indicated that these peptides presented identity with a ferric iron-catecholate outer membrane transporter (CirA) from *Mannheimia succiniciproducens* MBEL55E (accession number: YP_087708) and with a TonB-dependent receptor from *G. anatis* F149^T^ (accession number KGQ57895.1) (Supplementary data sheet 1). This result indicates that *G. anatis* could have the ability to uptake iron from siderophores.

The peptides obtained by MALDI-TOF analysis of the 70-kDa protein indicated that this protein was a TonB-dependent heme/hemoglobin receptor family member from *G. anatis* (access number: WP_013747074.1). A similar protein was also identified in OMP samples from *G. anatis* F114 and ESV34. This result indicates that this protein is conserved in other *G. anatis* strains and presents a similar response to iron stress (Figure 3A).

### Siderophore search

To determine whether *G. anatis*, in addition to getting siderophores, could also produce them, a spectrophotometric determination was performed. Spent media from *P. aeruginosa* presented a maximum absorption at approximately 400–450 nm in the presence or absence of 2,2′-dipyridyl; being higher in the presence of the chelating agent and suggesting the presence of siderophores; however, this change was not observed with spent media from *G. anatis*. A similar result was also observed in CAS-BHI agar. A yellow-orange halo was observed with *P. aeruginosa* strains, indicating the production of siderophores, but not around *G. anatis* or *E. coli* DH5α, which was used as a negative control (data not shown).

### *G. anatis fur* amplification

The possibility that the putative hemoglobin and siderophore receptors would be under positive or negative control by a Fur protein was answered by amplification, cloning, and interruption of the *G. anatis* 12656-12 *fur* gene. PCR amplification of the *G. anatis* 12656-12 locus *fur* gene gave a 2.2 kbp product containing a 593 bp *fur* gene flanked by the *fld*A and *mog*A genes in a similar manner as reported for *G. anatis* UMN179 (pSAMfur). The sequences of the *G. anatis* 12656-12 *fur* locus and *fur* gene share 97% similarity with those reported for the UMN179 strain (YP_004420910.1). pSAMfur was amplified by inverse PCR using primers GfurC2F and GfurCf to introduce a streptomycin cassette and interrupt the *fur* gene to generate pSAMΩfur (Figure 1A).

### *G. anatis fur* mutant generation

**FIGURE 4:**
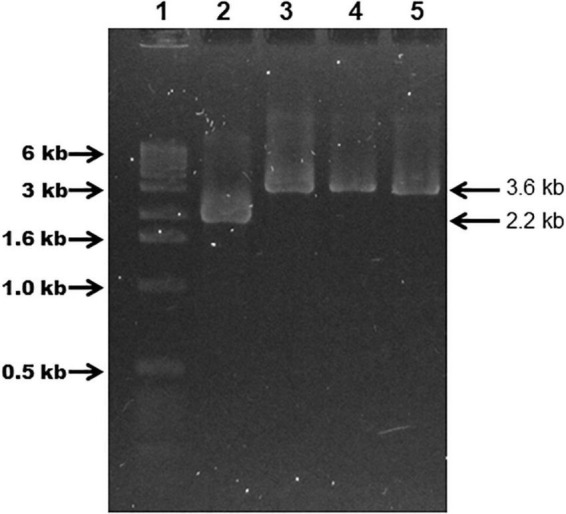
*G. anatis* 12656-12 (Ω *fur*) streptomycin-resistant mutants verified by PCR. Agarose gel (0.9%) showing 12656-12 *fur* WT (2.2 kbp band) and mutant *fur::sm* (3.6 kbp band) PCR amplification products obtained with *G. anatis* total DNA. (1) MWM, (2) *G. anatis* 12656-12, (3) construct pSAMΩfur in *E. coli*, (4) *G. anatis* Ω *fur* 126.3, (5) *G. anatis* Ω *fur* 126.5.

Plasmid pSAMΩfur was introduced into *G. anatis* 12656-12 by a M-IV chemical transformation step. *G. anatis* 12656-12-pSAMΩfur was cultured in the presence of streptomycin to select pSAMΩfur. Cells were plated on BHI agar plus streptomycin and patched on the same agar plates to identify colonies where homologous recombination had occurred. In total, 12 putative mutants were recovered that were streptomycin resistant. Five of these putative mutants were selected to verify that homologous recombination had occurred in the selected DNA locus and were manipulated *in vitro*. Chromosomal DNA from the parent, 12656-12 strain, and from five putative mutants, 1: Ω fur 126.13, 2: Ω fur 126.5, 3: Ω fur A1, 4: Ω fur A2, and 5: Ω fur B2, were analyzed by PCR. Primers specific to the *fur* locus amplified a 3.6-kbp product from the putative mutants and a 2.2 kbp product from the wild-type strain 12656-12 (Figure 4). The latter suggested that all five putative mutants had a *fur* gene interrupted by the streptomycin cassette.

### *G. anatis fur* mutant verification

**FIGURE 5:**
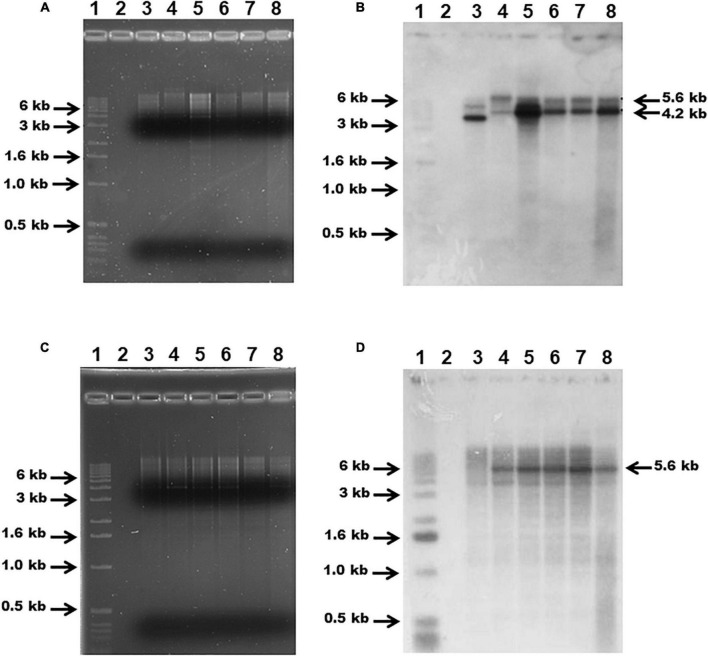
Agarose gel (1%) electrophoresis of *G. anatis* total DNA digested with *Hind*III **(A,C)** and DNA-DNA hybridization **(B,D)**: using *flavodoxin-fur* [2.2 kbp, **(B)**] or *streptomycin* [1.5 kbp, **(D)**] gene probes, respectively. (1) 1 kbp MWM, (2) empty, (3) *G. anatis* 12656-12 (wt). The mutants are in the following lines: (4) Ω *fur* 126.13, (5) Ω *fur* 126.5, (6) Ω *fur* A1, (7) Ω *fur* A2, (8) Ω f*ur* B2.

Chromosomal DNA from the wild-type *G. anatis* 12656-12 and the putative *fur* mutants was digested with *Hind*III enzyme, separated by agarose gel electrophoresis (Figures 5A,C), transferred onto a nylon membrane, and hybridized with *flavodoxin*-*fur* (Figure 5B) or streptomycin cassette (Figure 5D) probes. The *flavodoxin-fur* probe hybridized with the 5.6-kbp DNA band in the *G. anatis* mutants and a 4.2-kbp band in the wild-type strain (Figure 5B). The streptomycin probe hybridized only with DNA from the putative *G. anatis fur* mutants (Figure 5D). All these results confirmed that the *fur* locus had been interrupted and replaced in these strains.

### Putative siderophore receptor is under control of Fur

**FIGURE 6:**
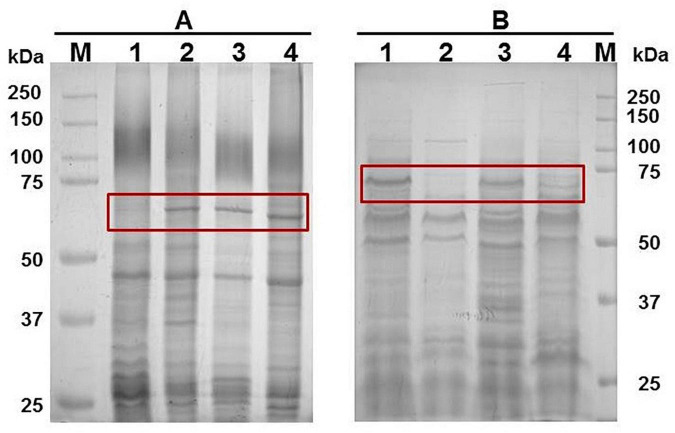
Ten percent polyacrylamide gel showing the 1% Triton-insoluble **(A)** or soluble **(B)** protein patterns of *G. anatis* 12656 WT (lanes 1 and 2) and Ω *fur* 126.13 (lanes 3 and 4) without (lanes 1 and 3) or with 2,2′-dipyridyl (lanes 2 and 4).

When the wild-type *G. anatis* 12656-12 and the 126.13 *fur* mutant were grown in the presence or absence of 2,2′-dipyridyl, a 65-kDa protein was expressed only in the presence of the chelating agent in the wild-type 12656-12 strain but was constitutively expressed in the Ω *fur* 126.13, in both conditions, confirming that the expression of the putative siderophore receptor is regulated by Fur (Figure 6A). However, expression of the 70-kDa protein diminished in the presence of 2,2′-dipyridyl in both wild-type 12656-12 and Ω *fur* 126.13 (Figure 6B), leading us to conclude that the putative hemoglobin-binding protein is not Fur controlled.

### Effect of FeCl_3_ on *G. anatis* growth and biofilm production

**FIGURE 7:**
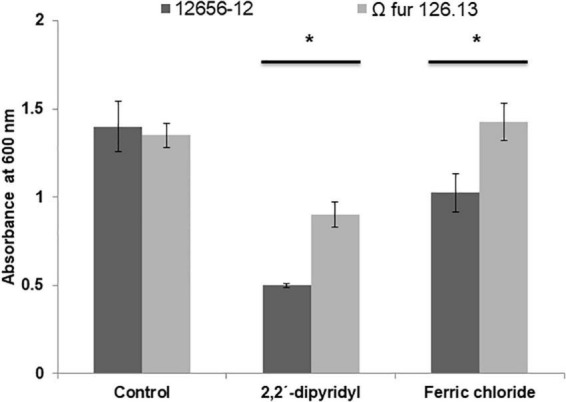
Growth of *G. anatis* 12656-12 and Ω *fur* 126.3 strains in 24-h cultures supplemented or not with 2,2′-dipyridyl or FeCl_3_. *Significant difference (*P* < 0.05), compared with the control.

**FIGURE 8:**
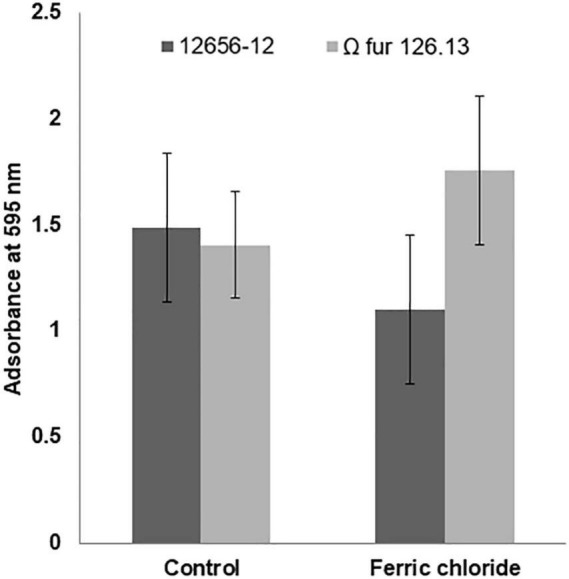
*G. anatis* 12656-12 and Ω *fur* 126.3 strains biofilm production in 24-h cultures supplemented or not with FeCl_3_.

The addition of FeCl_3_ to the 12656-12 strain culture medium diminished growth (approximately 30%) with respect to the culture without addition; however, the growth of Ω *fur* 126.13 was not affected (Figure 7). Also, in *G. anatis* 12656-12, presence of FeCl_3_ diminished biofilm production in 35%; in contrast, in the Ω *fur* 126.13, biofilm production increased in a similar percent (Figure 8), without a significant difference.

### *G. anatis* chicken inoculation

Chickens did not present any clinical signs along the 10 days post-inoculation. When re-isolation of *G. anatis* wild-type or Ω *fur* 126.13 strains were tried, 60% of the birds inoculated with the 126.13 strain were positive in heart samples; on the other hand, the wild-type strain was isolated in 20% of samples but from different organs (Table 1). *G. anatis* was not isolated from control group birds.

**TABLE 1:** Re-isolation of *G. anatis* 12656-12 and Ω *fur* 126.3 strains from SPF chickens intranasally inoculated.

		Post-infection re-isolation													
		126.13 (*fur*)							12656-12 (wt)						
	Chicken’s number	2	3	4	6	7	8	9	1′	3′	4′	6′	7′	8′	10′
	Windpipe									X					X
	Kidney										X		X		
Organ	Liver								X			X			
	Lung		X			X									
	Heart	X	X	X	X		X	X		X					X
	Infraorbital sinus												X	X	

Re-isolation of G. anatis strains were tested at 10 days post inoculation. Two ten-SPF-chickens groups were intranasal inoculated. Numbers indicated only the chickens with positive re-isolation. Re-isolation from air sacs, blood vessel was negative, and it is not shown.

## Discussion

Iron restriction is a non-specific host mechanism that limits the optimal conditions for the reproduction of microorganisms. However, most microorganisms have a repertoire of different strategies, considered as virulence factors, to obtain the necessary micronutrients. To survive in a host with restricted micronutrients, such as iron, pathogenic microorganisms must be able to withdraw iron bound by the host’s iron-binding proteins. Iron can be obtained by the production of low molecular weight molecules with high affinity to iron, called siderophores, or of outer membrane proteins with the capability to bind host transferrins or hemoproteins. In this work, the ability to overexpress proteins from *G. anatis* grown under iron-restricted conditions was evaluated. *G. anatis* grown in the presence of 2,2′-dipyridyl exhibited a reduction (60%) in its growth with respect to cultures with no addition of 2,2′-dipyridyl (Figure 7), similarly as described for *A. paragallinarum*, another *Pasteurellaceae* member that also infects chickens (Abascal et al., 2009).

*G. anatis* iron-restricted growth induced the up-expression of a putative siderophore receptor (65-kDa protein), and the down-expression of a putative hemoglobin receptor (70-kDa protein), both proteins were immune-recognized by sera of chickens infected with *G. anatis*, indicating their *in vivo* expression. However, although *G. anatis* expresses a putative siderophore receptor, this bacterium does not produce siderophores as was observed by spectrophotometric determinations and chromo azurol-BHI agar assays. This result was also supported by the absence of genes involved in siderophore synthesis within the *G. anatis* genome (NC_015460.1). However, *G. anatis* possess, besides a putative siderophore receptor, other proteins, like the iron ABC transporter permease (WP_013745286.1 and WP_013746863.1) and an FTR1 family iron permease protein (WP_013745724.1), which could be involved in the transport of siderophores produced by other microorganisms. It has been described that *Pasteurellaceae* members do not produce siderophores (Cornelissen, 2003), but that they contain the genetic components to use those produced by other microorganisms (Morton et al., 2010).

There is evidence that iron-uptake genes’ regulation is largely due to Fur proteins. Fur proteins function mainly by negative regulation; however, there is evidence indicating that Fur proteins can also act as transcriptional activators (Jin-Won and Helmann, 2007; Runyen-Janecky, 2013). In this case, it seems that both negative and positive actions of gene regulation could be exerted by Fur or that other bacterial gene expression regulators could be participating, with respect to the putative *G. anatis* siderophore and hemoglobin receptors, respectively. This effect can be observed in the gel (Figure 2). The putative siderophore receptor (65-kDa protein) was found at a higher concentration in samples of *G. anatis* grown in iron-restricted conditions, and lower in samples under normal conditions. In contrast, the putative hemoglobin-binding protein (70-kDa protein from the culture without the added chelating agent) was at a higher concentration in samples from the F149^T^ strain, and a tiny band was observed in iron-restricted conditions (Figure 4). *G. anatis* F149^T^ is a non-hemolytic and non-virulent strain with few studies on its participation in gallibacteriosis (Montes-García et al., 2016; Zhang et al., 2017). However, this *G. anatis* strain also expresses different virulence factors as virulent strains do, but in a lower amount. The higher expression of the putative hemoglobin-binding protein by *G. anatis* F149^T^ strain could be due to an incapacity to hemolyze red blood cells to get the necessary iron. A higher amount of this protein could ensure the supply of the necessary iron for this bacterium.

The constitutive expression of the putative *G. anatis* siderophore receptor in *G. anatis fur* mutants in the presence or not of 2,2′-dipyridyl indicates that this putative receptor is under control of the Fur protein, however, no changes were observed in the expression of the putative hemoglobin receptor in the *G. anatis* 12656-12 wild-type or its fur mutant, which indicates that the 70-kDa protein is not under Fur control. Besides the 70-kDa protein that interacts with the chicken biotinylated hemoglobin, two proteins of 35 kDa and 100 kDa also interact with hemoglobin (Figure 3B); but they will be the object of a future study.

**FIGURE 9:**
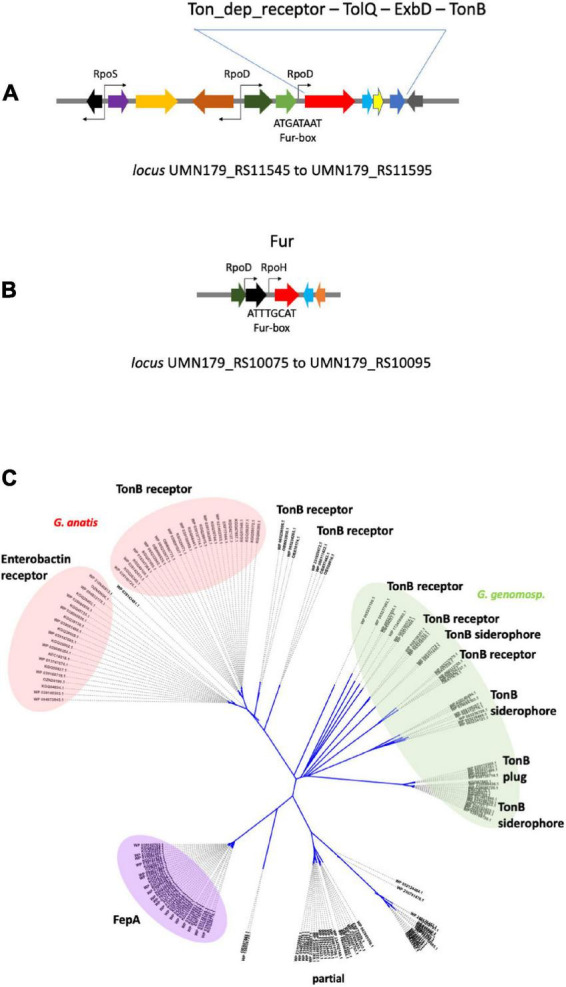
TonB-dependent receptors *in Gallibacterium* sp. **(A)** Map of the locus harboring the TonB-dependent receptor gene (FepAG) from *G. anatis* UMN179 chromosome. Gene encoding FepAG (boxed red arrow) is homologous to the enterobactin receptor gene (*erf77464.1*) and seems to be part of a four- members operon. The promoter nearest to fepAG was predicted as a RpoD-dependent promoter, indicated as RpoD above the tinny black arrow; a Fur-box with its consensus sequence was in the same intergenic region as indicated below the gray line. **(B)** Map of the locus encompassing the *fur* gene (red boxed arrow) in *G. anatis* UMN179 chromosome. The two promoters predicted to be close to *fur* are indicated as tinny black arrows in the drawing; one would depend on RpoH and the other on RpoD. One or both could control the gene expression of *fur*; below the proximal promoter, a Fur-box with its consensus sequence is indicated in the drawing. **(C)** Phylogeny of TonB-dependent receptors in the genus *Gallibacterium* sp. A total of 178 amino acid sequences were recovered from 41 sequenced genomes (GeneBank, Supplementary Information), of these: *G. anatis* (31 strains), *G. genomospecies* (7 strains), and *G. salpindigitis* (3 strains). An unrooted tree was built with the data; the phylogram shows five clades of proteins phylogenetically related to TonB-dependent receptor proteins from *G. anatis.* The first clade containing the protein from *G. anatis* UMN179 was termed enterobactin receptor (left, pink) because of the homology of proteins with the transferrin receptor sequence from *Shigella boidii* (accession EFX6075140.1). Another protein group (right, pink) was termed TonB receptor originating from *G. anatis* 12656-12. The disperse group, highlighted in green, encloses the TonB-dependent receptor and the TonB-dependent receptor of siderophores from *G. anatis, G. salpingitidis*, and *G. genomospecies.* The fourth clade, highlighted in purple, corresponds to receptors of the FepA-Like type. Clade marked as “partial” corresponds to incomplete sequence proteins of canonical receptors with b-barrel structure; the proteins of this group could correspond to the carboxyl-terminal domain of the TonB-dependent receptors.

Among bacteria from the *Pasteurellaceae* family several receptors have been implicated in the uptake of different sources of iron and annotated as proteins dependent on the TonB transport system (Whitby et al., 2009), i.e., the enterobactin, hemoglobin-transferrin, and siderophore receptors, in addition to others with unclear function (Lyles and Eichenbaum, 2018; Otero-Asman et al., 2019); moreover, along with these receptors, there is at least one that seems to be a pseudogene, as it is a truncated version of the aforementioned receptors containing only the carboxyl terminal sequence (accession ERF78509.1). In this work, we employed the *G. anatis* 12656-12 and F149^T^ strains as an experimental system, and the *G. anatis* UMN179 as a reference strain to analyze the behavior of the protein-receptors in a Fe^++^-deficient environment. *G. anatis* UMN179 is the only known strain whose circularized genome has been publicly released (accession number NC_015460.1 under Material and methods). In this work, the locus containing an operon-like arrangement was identified, where the enterobactin or hemoglobin transporter gene was located, along with the putative genes encoding the periplasmic proteins ExbB and ExbD, and the plug or stopper protein TonB. Gene organization of the predicted operon is conserved in a single copy in the three strains, as seems to be its function, so disturbances affecting our system could be minimal. Two other genes encoding additional hemoglobin or siderophore transporters were harbored in the examined chromosomes, but they are not a share of the *exbB, exbD, tonB* array. In the Figure 9A image, co-transcription of the operon is hypothesized on the basis of the gene’s organization and the finding of a predicted promoter and a RpoD binding site; reinforcing the idea regarding the identification of a putative Fur-box motif that could contribute to the iron-dependent regulation of the Fur protein and the operon, which could induce the TonB-dependent protein of 65 kDa in response to iron depletion. In contrast, the 70-kDa protein seems to be a membrane protein, whose expression is not induced by dipyridyl (Figure 2A, lane 4). At first sight, a postranscriptional mechanism regulates its disruption to supply the 65 kDa protein, which is recognized by the same antibodies as the 70-kDa isoform (Figure 2C). Possibly the 70-kDa protein is a precursor of the 65- kDa protein, its production could be a consequence of the protein processing favored by Fur de-repression, or from mechanical disruption, rather than a change in the use of the transcription start site (Duval et al., 2017, Grove, 2022). Mass spectrometry analysis showed that amino-terminal amino acids were maintained in both the 65-kDa and 70-kDa proteins, demonstrating the presence of both isoforms of the protein.

The Fur-box sequences were predicted further *in silico* by the Bprom program (Figures 9A,B), when Fur-box sequences from the TonB-dependent receptor gene and the *fur* gene were compared, despite that both are AT-rich they were not identical, this was previously observed when Fur- boxes in *fur* regulated genes also varied in sequence in other bacterial species that contain more complex Fur boxes. Affinity of *fur* for Fur-boxes in the TonB-dependent receptor and *fur* genes of *G. anatis* need to be further investigated.

A great variety of proteins resembling TonB-dependent receptors were grouped in the phylogenetic tree (Figure 9C) based on: (1) the substantial number of sequenced and released genomes of the *Gallibacterium* genus, either as draft or complete genome sequences, from which we knew many different types of TonB-dependent receptor proteins. (2) Proteins related to the TonB-dependent receptor were three per genome, one of which was a truncated gene (Figure 9C). All these proteins are considered outer membrane proteins, with conserved β-barrel-like structure, so it was difficult to infer which type of receptor/transporter they were and if they can uptake and transport different types of iron-containing molecules (Roumia et al., 2021). We do not know whether the iron needed for essential functions stimulated the same acquisition pathway in the bacterial genus nor the mechanism to avoid the excessive accumulation of Fe or Fe complexes. It seems this diversity of related proteins is maintained to contribute to coping with iron influx to prevent severe cell damage (Whitby et al., 2009; Neumann et al., 2017; Parker et al., 2022). In the phylogenetic study, we include proteins from complete CDSs or those whose domains were as long as their predicted and annotated proteins in the GenBank. The proteins grouped in the colored ovoids (Figure 9C) share sequence identities more significant than 85%, with E values greater than 5e-100, and more than 90% coverage. Differences between groups were less than 50%, with E values of 5e-50 and less than 80% coverage. Considering the values specified above for the 65- and 70-kDa proteins, the existence of a specific proteolytic process may alter the β-barrel structure and possibly the permeability of the transport channels from the TonB-dependent receptors also. This unrecorded observation of mobility change, size change, and structure change of TonB-dependent receptors needs further understanding. Likewise, it is necessary to study the possible biochemical and molecular processes that could generate TonB-dependent receptor isoforms (Duval et al., 2017; Roumia et al., 2021; Parker et al., 2022). It is tempting to think of the DegP-type proteases from periplasmic spaces or external membranes as potential candidates (Ge et al., 2014; Gómez-Santos et al., 2019).

Other important regulators could be considered to control OMP changes, for example the general stress response regulator RpoS (Wang et al., 2014), and MarR, which controls the expression of genes involved in various adaptive molecular mechanisms, such as antibiotic and antimicrobial stress resistance, oxidative stress response, heat shock resistance and virulence, leading to transcriptional repression or activation (Wilkinson and Grove, 2006). The identification of the right/specific gene regulator of the 70-kDa protein and a wider characterization of the *G. anatis* 126.13 *fur* mutant will be performed in future works.

In *Xanthomonas campestris*, the *fur* gene mutation induces a growth reduction due to a high siderophore and iron transport genes expression (Jittawuttipoka et al., 2010). The addition of FeCl_3_ to the culture medium induced a diminishing growth of the wild-type *G. anatis*, suggesting a toxic effect, probably due to a Fenton reaction. However, the same addition did not affect the growth of the Ω *fur* 126.13 strain indicating Fur-independent regulator mechanisms in *G. anatis*. Among these mechanisms could be the anti-porter systems that expel the excess of iron to the cell’s exterior as described for *Streptococcus suis* (Jia et al., 2020). Another mechanism could be the up- expression of different antioxidant enzymes, such as the superoxide dismutase enzyme. In *Stenotrophomonas maltophilia*, it has been described that MnSOD increases activity in *fur* mutants, in comparison with strain type, indicating that MnSOD is negatively regulated by the *fur* gene (García et al., 2015). To understand this behavior, more experiments are needed.

In *Staphylococcus aureus* and *Stenotrophomonas maltophilia*, biofilm formation diminishes in the presence of iron and increases in iron-restriction conditions (Johnson et al., 2005; García et al., 2015). In *S. maltophilia*, a higher biofilm production was described by a spontaneous *fur* mutant, similarly as observed herein with the *G. anatis fur* mutant. This higher amount in *S. maltophilia* biofilm production was due to an increase in exopolymeric components produced by the *fur* mutant that were no different in composition from those produced by the wild-type strain (García et al., 2015). In *Yersinia pestis*, an alternative mechanism has been described in which Fur inhibits biofilm formation by repressing *hms*T and, in consequence, inhibiting c-di-GMP synthesis (Porcheron and Dozois, 2015). High levels of c-di-GMP have been related with biofilm formation, and vice versa. The mechanisms involved in these effects in *G. anatis* will be considered in future work.

Interruption of the *fur* gene in *G. anatis* seems to diminish its pathogenic capacity because Ω *fur* 126.13 was only re-isolated from heart samples, whereas the wild-type strain was isolated, albeit at a lower percentage, but from different organs (Table 1). The reason about colonization mainly of heart tissues is unknown. Relevance of Fur in bacterial infections is different depending on the bacterial species and model of infection (Pasqua et al., 2017). The ability to count with different iron mechanisms could be an advantage for a pathogen microorganism, such as *G. anatis*.

## Data availability statement

The original contributions presented in this study are included in the article/Supplementary material, further inquiries can be directed to the corresponding author.

## Ethics statement

The animal study was reviewed and approved by Científicos Veterinarios de México. Av. Luis Donaldo Colosio 984-6, Tepatitlán, Jalisco, Mexico. Written informed consent was obtained from the owners for the participation of their animals in this study.

## Author contributions

AC-G, SM-P, NR-R, IR-H, FM-G, HT-R, and IY-A performed the experiments. AC-G contributed to the microbiology, physiology, and genetics. SM-P contributed to the genetics. NR-R and IY-A contributed to the microbiology and physiology. IR-H contributed to the immunology. FM-G and HT-R contributed to pathogenicity. CV-C, PS-A, and EN-A made revision of results and data analysis and provided the funding. CV-C and EN-A wrote the manuscript. All authors critically reviewed, edited, and approved this work.

## Footnotes

1
www.ncbi.nlm.nih.gov

2
www.ebi.a-c.uk/Tools/clustalw2/index.html

3
http://www.softberry.com/berry.phtml?topic=bprom&group= programs&subgroup=gfindb

4
https://blast.ncbi.nlm.nih.gov/Blast.cgi

5
https://www.ebi.ac.uk/Tools/msa/clustalo/

6
https://itol.embl.de/

7
https://www.rcsb.org/

8
https://swissmodel.expasy.org/

9
http://www.matrixscience.com/server.html

## Abbreviations

BHI: brain heart infusion
CAS: chrome azurol S
CFU: colony-forming units
Fur: ferric uptake regulator
OMP: outer membrane protein
PCR: polymerase chain reaction
RTX: repeats in toxins
SDS-PAGE: sodium dodecyl sulfate-polyacrylamide gel electrophoresis.
