# Supplementary material for: A putative siderophore receptor of Gallibacterium anatis 12656-12 under Fur control also binds hemoglobin
Source: Front Microbiol. 2022 Aug 16;13:951173. doi: 10.3389/fmicb.2022.951173 (PMC9425032; doi:10.3389/fmicb.2022.951173)
Supplement: Supplementary file 1 [file Data_Sheet_1.PDF]

Putative siderophore receptor

Mannheimia succiniciproducens TonB-dependent receptor (WP_011199695.1)

MKKLKISLLPLTAFVAATVHAETLDTIDVVSDNFSPQAENIAAKGVTKVRQATKMSDVIRGV**PGVNVNGAR**S
TVERYNIR**GVSEEYLNVTVDGAR**QNGYSFHHNGNYGIDPEILKRVDIDVGSNSVSTGAGSLGGSMKFETVDA
ADMLEEGENFGGKVKYGYGSNGNSNQGTAMLYGRRGNLDLLGYFNYRHQRDGEDGNGLKNKNK**GHLSNYLF**T
KYNISNEQWIKASAERYTNTALSCYRANMGMCLGDVPQPGEPGYVETNHGKAYTELTRKTYTLSYGFNPEHNN
WVNIKANAYNTETEVASMGSPKSKVRTVGGTLSNTSEFELGVTSHQFLVGGEYYNSKAQALGSVNNAYVADMD
STSVYYVEDKIALGNLMIIPGVRFDHYKADLASDFDKSYHRFSKALGLKYSLTDNLIVFANYTELFK**GPDAGI**
YLRGTRAYDGNLEAARGDNKEVGFSYAKDGLFSDIDGFSFTAKYFKTDYDNINQTVSASRCVNTSAISSGSIY
CNLGKVDIKGVEAQAKYRYEDTSFSVSYARARSEQKSTGLAAFADTGDRYNFTLSQYISSAQVELGWNTMYVR
AIDVDDSTLKESYAVSNMYVSWSPAQAKGLELTFGIDNIFDKAYKDHS TQYYGSVDLDPGRNYKLSVSYKF

Gallibacterium anatis DSM 16844 = F 149^{T} TonB-dependent receptor (KGQ57895.1)
MKKTSFVLLPLAAFISGVVVADEADNLDAIEVVSDNLSPQKASLSAGSLAKVRQATTTADILRSV**PGVNVNG**
ARSIVQRYSIR**GVSEEYLTVTVDGAR**QNGYAFHHAGNYGIDPDILKRVNVDVGANSVVTGAGSLGGAIRFG
TVEAADLLADGENFGAKFKWGYGSSADSNQAATTLYGRVGGLDVLGYFNYRHQENGKDGNGVENANK**GHLSNY**
LFKAKYNIDDTQWVKFSAERYNNTALSCYRANFNFCLGDVPKPGEQGYNETNHGKALTELLRKTYTLSYGIDP
KDNPYLNFKANLYNTETDMSSMGKPQTNIRTVGGTVSNIAEIDLANTHHSITFGGEYYNTKSRALNAPDKSYQ
PRIDSTSVYLEDKIAVGDFLITPGVRYDYYQADLDKNFDKSYQRFSKALGLKYLLTDDLAVFANYTEIFK**GPD**
AGEIYLRGTRNYLPSIDAIRGNNKEAGMSLAKADIFGNDDFSLTAKYFQTKYNHFNTNTITEQGSGYQDIGE
VKVKGVEVSTAYRINNLFINAGYARARSEQLDPLFGFYNLTALPDTGDKYTLGLSYVMPDYGVELGWNTIWVR
SITINSAEKRGGPAKKTETYKESYSVSNVYATWSPKQLPSLELTAGIDNIFDKAYKDQATKYYAASDNELGRN
YKFTVSYKF

Hemoglobin binding protein

Gallibacterium anatis TonB-dependent receptor (WP_013747074.1)

MKKTSFVLLPLAAFISGVVVADETDNLDAIEVVSDNLSPQKASLSAGSLAK**VRQATTTADILR**SVPGVNVNG
ARSVVQRYSIR**GVSEEYLTVTVDGAR**QNGYAFHHAGNYGIDPDILKR**VNVDVGANSVVTGAGSLGGAIR**
FETVEAADLLADGENFGAK**FKWGYGSNADSNQAATTLYGR**AGGLDVLGYFNYRHQENGKDGNGVENANKGH
LSNYLFK**AKYNIDEAQWVK**FSAERYDNTALSCATANFACAENSEKKLAGILRKTYTVAYGYAPSDNPLLNVK
ANFYNTKTVVSAMDADKSRIRTVGGTLSNVSEIDIVNTHHSITVGGEYYSTKSLALGARPDQRIKNFYDASVD
STSIYLEDKIAVGDFIITPGVRYDYYQADLSKDFDKSYKRFSKALGLKYLLTDDLAIFANYTEIFKGPDVGEV
FLSTIPTNYHPSIDAIRGNNKEAGFSFVK**ADIFGNDDFSLTAK**YFQTKYNHFNTNIESERSTGSAYQDIGEV
K**VKGVEVSTAYRINNFSINAGYAR**ARS**EQLDPLFGFYNLTALPDTGDK**YTLGLSYAVPDYGIELGWNTI
WVRSITVDSAGRKDRTKNSPIVKKETYKESYSVSNVYATWSPKQLPNLELTAGIDNIFDKAYVDQATKYYSTA
YPSSENRYIGGDYELGR**NYKFTVSYKF**
