# Supplementary material for: A putative siderophore receptor of Gallibacterium anatis 12656-12 under Fur control also binds hemoglobin
Source: Front Microbiol. 2022 Aug 16;13:951173. doi: 10.3389/fmicb.2022.951173 (PMC9425032; doi:10.3389/fmicb.2022.951173)
Supplement: Supplementary file 2 [file Data_Sheet_2.PDF]

Locus_tags for genes from *G anatis* UMN179 and 12656-12 strains and proteins encoded

TonB dependent receptor locus

UMN179	locus_tag	protein	12656-12	locus_tag	protein
chromosome	UMN179_RS11545	WP_013747067.1	contig	N561_11290	ERF77458
NC_015460.1	UMN179_RS11550	WP_013747068.1	AVOX01000040	N561_11295	ERF77459
	UMN179_RS11555	WP_013747069.1		N561_11300	ERF77460
	UMN179_RS11560	WP_013747070.1		N561_11305	ERF77461
	UMN179_RS11565	WP_013747071.1		N561_11310	ERF77462
	UMN179_RS11570	WP_013747072.1		N561_11315	ERF77463
	UMN179_RS11575	WP_013747074.1		N561_11320	ERF77464
	UMN179_RS11580	WP_013747075.1		N561_11325	ERF77465
	UMN179_RS11585	WP_013747076.1		N561_11330	ERF77466
	UMN179_RS11590	WP_013747077.1		N561_11335	ERF77473
	UMN179_RS11595	WP_013747078.1		N561_11340	ERF77467

Fur locus

UMN179	locus_tag	protein	12656-12	locus_tag	protein
chromosome	UMN179_RS10075	WP_013746769.1	contig	N561_08725	ERF77928
NC_015460.1	UMN179_RS10080	WP_013746770.1	AVOX01000062	N561_08730	ERF77929
	UMN179_RS10085	WP_026211122.1		N561_08735	ERF77930
	UMN179_RS10090	WP_013746772.1		N561_08740	ERF77931
	UMN179_RS10095	WP_013746773.1		N561_08745	ERF77932
