# Supplementary material for: A putative siderophore receptor of Gallibacterium anatis 12656-12 under Fur control also binds hemoglobin
Source: Front Microbiol. 2022 Aug 16;13:951173. doi: 10.3389/fmicb.2022.951173 (PMC9425032; doi:10.3389/fmicb.2022.951173)
Supplement: Supplementary file 3 [file Data_Sheet_3.PDF]

Proteins included in phylogenetic tree analysis

AEC18276.1 outer membrane receptor FepA [Gallibacterium anatis UMN179]
AEC18318.1 enterobactin receptor protein [Gallibacterium anatis UMN179]
ERF77464.1 TonB-dependent receptor [Gallibacterium anatis 12656/12]
ERF78176.1 ligand-gated channel protein [Gallibacterium anatis 12656/12]
ERF78217.1 hypothetical protein N561_07380 [Gallibacterium anatis 12656/12]
ERF78509.1 hypothetical protein N561_06045 [Gallibacterium anatis 12656/12]
KGQ26340.1 TonB-dependent receptor [Gallibacterium anatis CCM5995]
KGQ26582.1 outer membrane receptor protein [Gallibacterium anatis]
KGQ26776.1 outer membrane receptor protein [Gallibacterium anatis]
KGQ28615.1 TonB-dependent receptor [Gallibacterium anatis]
KGQ29455.1 TonB-dependent receptor [Gallibacterium anatis]
KGQ29571.1 TonB-dependent receptor [Gallibacterium anatis]
KGQ30712.1 CirA protein [Gallibacterium anatis]
KGQ30744.1 TonB-dependent receptor [Gallibacterium genomosp. 2]
KGQ31244.1 TonB-dependent receptor [Gallibacterium genomosp. 2]
KGQ31679.1 outer membrane receptor protein [Gallibacterium anatis]
KGQ31821.1 ligand-gated channel protein [Gallibacterium genomosp. 2]
KGQ32668.1 outer membrane receptor protein [Gallibacterium genomosp. 2]
KGQ33816.1 TonB-dependent receptor [Gallibacterium anatis]
KGQ35002.1 TonB-dependent receptor [Gallibacterium anatis]
KGQ38059.1 TonB-dependent receptor [Gallibacterium anatis IPDH697-78]
KGQ38793.1 TonB-dependent receptor [Gallibacterium genomosp. 1]
KGQ38928.1 outer membrane receptor protein [Gallibacterium genomosp. 1]
KGQ39136.1 TonB-dependent receptor [Gallibacterium anatis]
KGQ42137.1 TonB-dependent receptor [Gallibacterium anatis]
KGQ43031.1 outer membrane receptor protein [Gallibacterium anatis]
KGQ44791.1 outer membrane receptor protein [Gallibacterium anatis]
KGQ45735.1 TonB-dependent receptor [Gallibacterium anatis]
KGQ47453.1 outer membrane receptor protein [Gallibacterium anatis]
KGQ47957.1 TonB-dependent receptor [Gallibacterium anatis]
KGQ48619.1 outer membrane receptor protein [Gallibacterium anatis]
KGQ49100.1 TonB-dependent receptor, partial [Gallibacterium anatis 10672-6]
KGQ49617.1 ligand-gated channel protein [Gallibacterium anatis]
KGQ50753.1 outer membrane receptor protein [Gallibacterium anatis 10672-6]
KGQ51548.1 TonB-dependent receptor [Gallibacterium anatis]
KGQ54579.1 ligand-gated channel protein [Gallibacterium anatis str. Avicor]
KGQ55927.1 TonB-dependent receptor [Gallibacterium anatis str. Avicor]
KGQ55955.1 outer membrane receptor protein [Gallibacterium anatis str. Avicor]
KGQ57521.1 outer membrane receptor protein [Gallibacterium anatis]
KGQ57895.1 TonB-dependent receptor [Gallibacterium anatis DSM 16844 = F 149]
KGQ58257.1 TonB-dependent receptor [Gallibacterium anatis]
KGQ58572.1 TonB-dependent receptor [Gallibacterium anatis 4895]
KGQ63184.1 outer membrane receptor protein [Gallibacterium anatis 4895]
KGQ63207.1 outer membrane receptor protein [Gallibacterium anatis]
KGQ64441.1 TonB-dependent receptor [Gallibacterium anatis]
KGQ64796.1 outer membrane receptor protein [Gallibacterium anatis 7990]
KGQ64834.1 TonB-dependent receptor [Gallibacterium anatis 7990]
KGQ65169.1 ligand-gated channel protein [Gallibacterium anatis]
KGQ66007.1 TonB-dependent receptor [Gallibacterium anatis]
KGQ67845.1 ligand-gated channel protein [Gallibacterium anatis]
KGQ68285.1 TonB-dependent receptor [Gallibacterium anatis]
OBW92137.1 TonB-dependent receptor [Gallibacterium salpingitidis]
OBW93930.1 TonB-dependent receptor [Gallibacterium genomosp. 3]
OBW93962.1 TonB-dependent receptor [Gallibacterium genomosp. 3]
OBW96520.1 TonB-dependent receptor [Gallibacterium anatis]
OBW96775.1 TonB-dependent receptor [Gallibacterium anatis]
OBW97969.1 TonB-dependent receptor [Gallibacterium genomosp. 1]
OBW98128.1 TonB-dependent receptor [Gallibacterium genomosp. 1]
OBX03538.1 TonB-dependent receptor [Gallibacterium genomosp. 3]
OBX04257.1 TonB-dependent receptor [Gallibacterium genomosp. 3]
OBX04942.1 outer membrane receptor protein, partial [Gallibacterium salpingitidis]
OBX05418.1 TonB-dependent receptor [Gallibacterium genomosp. 3]
OBX07442.1 TonB-dependent receptor [Gallibacterium salpingitidis]
OBX07848.1 hypothetical protein QV08_06355, partial [Gallibacterium salpingitidis]
OBX08030.1 TonB-dependent receptor [Gallibacterium salpingitidis]
OBX08152.1 TonB-dependent receptor [Gallibacterium salpingitidis]
OBX09616.1 TonB-dependent receptor [Gallibacterium salpingitidis]
OBX10174.1 TonB-dependent receptor [Gallibacterium genomosp. 3]
OBX10623.1 TonB-dependent receptor [Gallibacterium genomosp. 3]

OBX11268.1 TonB-dependent receptor [Gallibacterium salpingitidis]
OBX11679.1 TonB-dependent receptor [Gallibacterium salpingitidis]
OZN26131.1 TonB-dependent receptor [Gallibacterium anatis]
OZN26199.1 TonB-dependent receptor [Gallibacterium anatis]
OZN27761.1 TonB-dependent siderophore receptor [Gallibacterium anatis]
OZN48242.1 TonB-dependent receptor [Gallibacterium anatis]
OZN49696.1 TonB-dependent receptor [Gallibacterium anatis]
WP_013745923.1 TonB-dependent siderophore receptor [Gallibacterium anatis]
WP_013747032.1 TonB-dependent hemoglobin/transferrin/lactoferrin family receptor [Gallibacterium anatis]
WP_013747074.1 TonB-dependent receptor [Gallibacterium anatis]
WP_018345523.1 TonB-dependent receptor [Gallibacterium anatis]
WP_018347364.1 TonB-dependent receptor [Gallibacterium anatis]
WP_021461824.1 TonB-dependent hemoglobin/transferrin/lactoferrin family receptor [Gallibacterium anatis]
WP_021461848.1 TonB-dependent siderophore receptor [Gallibacterium anatis]
WP_021462353.1 TonB-dependent receptor [Gallibacterium anatis]
WP_039081490.1 TonB-dependent receptor [Gallibacterium anatis]
WP_039084303.1 TonB-dependent siderophore receptor [Gallibacterium anatis]
WP_039084936.1 TonB-dependent receptor [Gallibacterium anatis]
WP_039086725.1 TonB-dependent siderophore receptor [Gallibacterium anatis]
WP_039087497.1 TonB-dependent hemoglobin/transferrin/lactoferrin family receptor [Gallibacterium anatis]
WP_039089354.1 TonB-dependent receptor [Gallibacterium anatis]
WP_039090351.1 TonB-dependent hemoglobin/transferrin/lactoferrin family receptor [Gallibacterium anatis]
WP_039091037.1 TonB-dependent receptor [Gallibacterium anatis]
WP_039092387.1 TonB-dependent hemoglobin/transferrin/lactoferrin family receptor [Gallibacterium anatis]
WP_039095038.1 TonB-dependent receptor [Gallibacterium anatis]
WP_039095562.1 TonB-dependent hemoglobin/transferrin/lactoferrin family receptor [Gallibacterium anatis]
WP_039134949.1 TonB-dependent hemoglobin/transferrin/lactoferrin family receptor [Gallibacterium genomosp. 2]
WP_039135714.1 TonB-dependent receptor [Gallibacterium genomosp. 2]
WP_039136248.1 TonB-dependent receptor [Gallibacterium genomosp. 2]
WP_039137714.1 TonB-dependent receptor [Gallibacterium anatis]
WP_039138736.1 TonB-dependent hemoglobin/transferrin/lactoferrin family receptor [Gallibacterium anatis]
WP_039141468.1 TonB-dependent hemoglobin/transferrin/lactoferrin family receptor [Gallibacterium anatis]
WP_039142431.1 TonB-dependent receptor, partial [Gallibacterium anatis]
WP_039143481.1 TonB-dependent receptor plug domain-containing protein, partial [Gallibacterium anatis]
WP_039146494.1 TonB-dependent siderophore receptor [Gallibacterium anatis]
WP_039147085.1 TonB-dependent receptor [Gallibacterium anatis]
WP_039148750.1 TonB-dependent siderophore receptor [Gallibacterium anatis]
WP_039149234.1 TonB-dependent hemoglobin/transferrin/lactoferrin family receptor [Gallibacterium anatis]
WP_039149303.1 TonB-dependent receptor [Gallibacterium anatis]
WP_039158959.1 TonB-dependent receptor [Gallibacterium anatis]
WP_039159394.1 TonB-dependent hemoglobin/transferrin/lactoferrin family receptor [Gallibacterium anatis]
WP_039160726.1 TonB-dependent receptor [Gallibacterium anatis]
WP_039161699.1 TonB-dependent siderophore receptor [Gallibacterium anatis]
WP_039162782.1 TonB-dependent hemoglobin/transferrin/lactoferrin family receptor [Gallibacterium anatis]
WP_039163433.1 TonB-dependent siderophore receptor [Gallibacterium anatis]
WP_039166851.1 TonB-dependent hemoglobin/transferrin/lactoferrin family receptor [Gallibacterium anatis]
WP_039168718.1 TonB-dependent receptor [Gallibacterium anatis]
WP_039169730.1 TonB-dependent siderophore receptor [Gallibacterium anatis]
WP_039171342.1 TonB-dependent hemoglobin/transferrin/lactoferrin family receptor [Gallibacterium genomosp. 1]
WP_039172300.1 TonB-dependent receptor plug domain-containing protein [Gallibacterium genomosp. 1]
WP_042907618.1 TonB-dependent hemoglobin/transferrin/lactoferrin family receptor [Gallibacterium anatis]
WP_052121907.1 TonB-dependent receptor [Gallibacterium anatis]
WP_052124457.1 TonB-dependent receptor [Gallibacterium anatis]
WP_052124484.1 TonB-dependent receptor [Gallibacterium anatis]

WP_052125190.1 TonB-dependent receptor [Gallibacterium anatis]
WP_065230574.1 TonB-dependent hemoglobin/transferrin/lactoferrin family receptor [Gallibacterium genomosp. 1]
WP_065230740.1 TonB-dependent hemoglobin/transferrin/lactoferrin family receptor [Gallibacterium genomosp. 1]
WP_065231489.1 TonB-dependent receptor [Gallibacterium genomosp. 1]
WP_065232672.1 TonB-dependent receptor [Gallibacterium anatis]
WP_065232942.1 TonB-dependent receptor [Gallibacterium anatis]
WP_065233151.1 TonB-dependent siderophore receptor [Gallibacterium genomosp. 1]
WP_065233356.1 TonB-dependent hemoglobin/transferrin/lactoferrin family receptor [Gallibacterium genomosp. 1]
WP_065234121.1 TonB-dependent siderophore receptor [Gallibacterium genomosp. 3]
WP_065234285.1 TonB-dependent receptor [Gallibacterium genomosp. 3]
WP_065236728.1 TonB-dependent siderophore receptor [Gallibacterium genomosp. 3]
WP_065236793.1 TonB-dependent receptor [Gallibacterium genomosp. 3]
WP_065237180.1 TonB-dependent hemoglobin/transferrin/lactoferrin family receptor [Gallibacterium genomosp. 3]
WP_065237605.1 TonB-dependent receptor [Gallibacterium genomosp. 3]
WP_065237979.1 TonB-dependent receptor [Gallibacterium genomosp. 3]
WP_065237993.1 TonB-dependent receptor [Gallibacterium genomosp. 3]
WP_065238486.1 TonB-dependent siderophore receptor [Gallibacterium genomosp. 3]
WP_065238550.1 TonB-dependent receptor [Gallibacterium genomosp. 3]
WP_065238556.1 TonB-dependent receptor [Gallibacterium genomosp. 3]
WP_066106357.1 TonB-dependent receptor [Gallibacterium salpingitidis]
WP_066107779.1 TonB-dependent siderophore receptor [Gallibacterium salpingitidis]
WP_066109297.1 TonB-dependent siderophore receptor [Gallibacterium salpingitidis]
WP_066111422.1 TonB-dependent receptor [Gallibacterium salpingitidis]
WP_066111695.1 TonB-dependent siderophore receptor [Gallibacterium salpingitidis]
WP_066112755.1 TonB-dependent receptor [Gallibacterium salpingitidis]
WP_066112776.1 TonB-dependent siderophore receptor [Gallibacterium salpingitidis]
WP_066114265.1 TonB-dependent receptor, partial [Gallibacterium salpingitidis]
WP_066421157.1 TonB-dependent receptor [Gallibacterium salpingitidis]
WP_082985556.1 TonB-dependent receptor [Gallibacterium genomosp. 1]
WP_084598488.1 TonB-dependent receptor [Gallibacterium genomosp. 2]
WP_094873179.1 TonB-dependent receptor [Gallibacterium anatis]
WP_094873845.1 TonB-dependent receptor [Gallibacterium anatis]
WP_094873929.1 TonB-dependent hemoglobin/transferrin/lactoferrin family receptor [Gallibacterium anatis]
WP_094933178.1 TonB-dependent receptor [Gallibacterium anatis]
WP_155759665.1 hypothetical protein [Gallibacterium anatis]
WP_156536729.1 TonB-dependent receptor, partial [Gallibacterium salpingitidis]
WP_172459082.1 MULTISPECIES: TonB-dependent hemoglobin/transferrin/lactoferrin family receptor [Pasteurellaceae]
WP_210640813.1 TonB-dependent receptor [Gallibacterium anatis]
WP_210641520.1 TonB-dependent hemoglobin/transferrin/lactoferrin family receptor [Gallibacterium anatis]
WP_230585763.1 TonB-dependent receptor [Gallibacterium anatis]
WP_230586433.1 TonB-dependent receptor [Gallibacterium anatis]
WP_230587033.1 TonB-dependent receptor [Gallibacterium anatis]
WP_230589087.1 TonB-dependent receptor [Gallibacterium anatis]
WP_230589122.1 TonB-dependent receptor [Gallibacterium anatis]
WP_230589436.1 TonB-dependent receptor [Gallibacterium anatis]
WP_230591789.1 TonB-dependent receptor [Gallibacterium anatis]
WP_230592124.1 TonB-dependent receptor [Gallibacterium anatis]
WP_230781476.1 TonB-dependent receptor [Gallibacterium anatis]
WP_231128983.1 TonB-dependent receptor [Gallibacterium anatis]
WP_231923672.1 TonB-dependent receptor [Gallibacterium salpingitidis]
WP_234702149.1 TonB-dependent receptor [Gallibacterium genomosp. 1]
WP_237699455.1 TonB-dependent receptor [Gallibacterium anatis]
WP_245176457.1 TonB-dependent receptor [Gallibacterium genomosp. 2]
WP_245176458.1 TonB-dependent hemoglobin/transferrin/lactoferrin family receptor [Gallibacterium genomosp. 2]
WP_246874706.1 TonB-dependent receptor [Gallibacterium anatis]
