# Supplementary material for: A putative siderophore receptor of Gallibacterium anatis 12656-12 under Fur control also binds hemoglobin
Source: Front Microbiol. 2022 Aug 16;13:951173. doi: 10.3389/fmicb.2022.951173 (PMC9425032; doi:10.3389/fmicb.2022.951173)

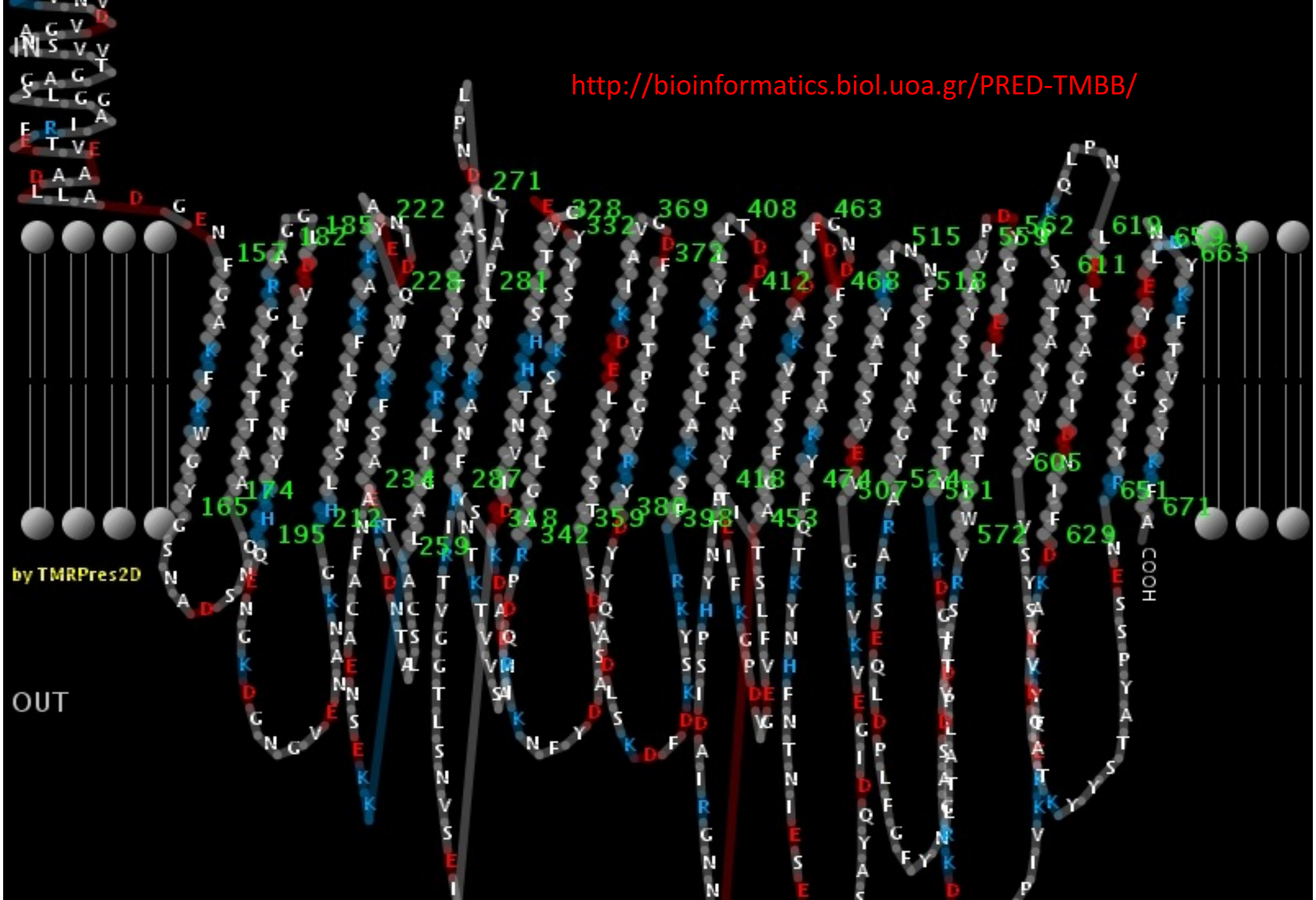
http://bioinformatics.biol.uoa.gr/PRED-TMBB/
by TMRPres2D
OUT
COOH

671 aa

```
MKKTSFVLLPLAAFISGVVVADETDNLDAIEVVSDNLSPQKASLSAGSLAKVRQATTTADILRSVPGVNVNGARSVVQRYSIRGVSEEYLTVTVDGARQNGYAFHHAGNYGIDPDILKRVNVDVG
1        10        20        30        40        50        60        70        80        90        100       110       120

ANSVVTGAGSLGGAIRFETVEAADLLADGENFGAKFKWGYGSNADSNQAATTLYGRAGGLDVLGYFNYRHQENGKDGNGVENANKGHLSNYLFKAKYNIDEAQWVKFSAERYDNTALSCATANFA
    130       140       150       160       170       180       190       200       210       220       230       240       250

CAENSEKKLAGILRKTYTVAYGYAPSDNPLLNVKANFYNTKTVVSAMDADKSRIRTVGGTLSNVSEIDIVNTHHSITVGGEYYSTKSLALGARPDQRIKNFYDASVDSTSIYLEDKIAVGDFIIT
         260       270       280       290       300       310       320       330       340       350       360       370

PGVRYDYYQADLSKDFDKSYKRFSKALGLKYLLTDDLAIFANYTEIFKGPDVGEVFLSTIPTNYHPSIDAIRGNNKEAGFSFVKADIFGNDDFSLTAKYFQTKYNHFNTNIESERSTGSAYQDIG
    380       390       400       410       420       430       440       450       460       470       480       490       500

EVKVKGVEVSTAYRINNFSINAGYARARSEQLDPLFGFYNLTALPDTGDKYTLGLSYAVPDYGIELGWNTIWVRSITVDSAGRKDRTKNSPIVKKETYKESYSVSNVYATWSPKQLPNLELTAGI
         510       520       530       540       550       560       570       580       590       600       610       620

DNIFDKAYVDQATKYYSTAYPSSENRYIGGDYELGRNYKFTVSYKF
    630       640       650       660       670 671
```

http://bioinformatics.biol.uoa.gr/PRED-TMBB/

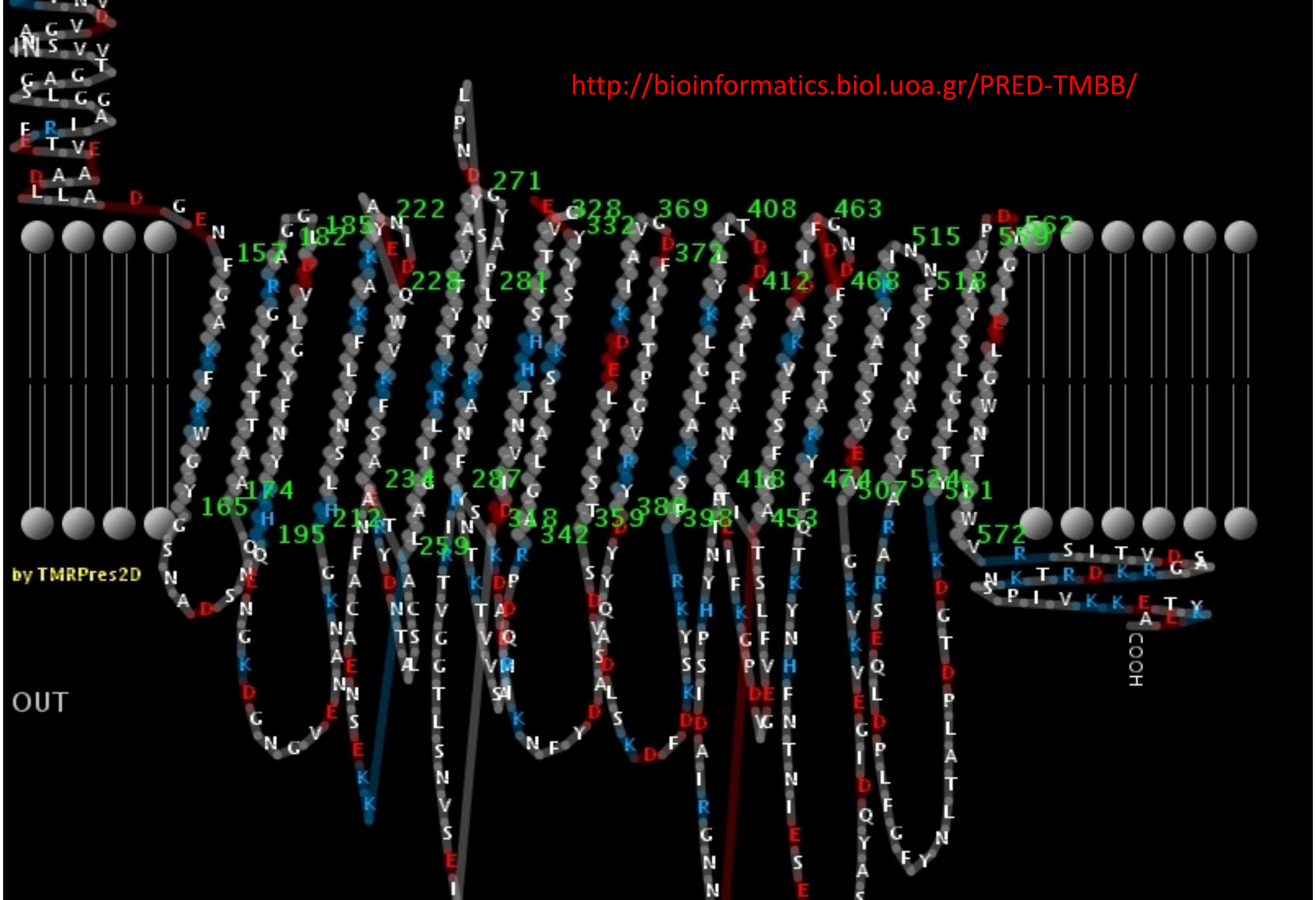

Supplement: Supplementary file 4 [file Data_Sheet_4.PDF]
